# Vegetable glycerin e-cigarette aerosols cause airway inflammation and ion channel dysfunction

**DOI:** 10.3389/fphar.2022.1012723

**Published:** 2022-09-26

**Authors:** Michael D. Kim, Samuel Chung, John S. Dennis, Makoto Yoshida, Carolina Aguiar, Sheyla P. Aller, Eliana S. Mendes, Andreas Schmid, Juan Sabater, Nathalie Baumlin, Matthias Salathe

**Affiliations:** ^1^ Department of Internal Medicine, Division of Pulmonary, Critical Care and Sleep Medicine, University of Kansas Medical Center, Kansas City, KS, United States; ^2^ Department of Medicine, Division of Pulmonary, Critical Care and Sleep Medicine, University of Miami Miller School of Medicine, Miami, FL, United States; ^3^ Department of Research, Mount Sinai Medical Center, Miami Beach, FL, United States

**Keywords:** e-cigarette, vegetable glycerin, CFTR, Mucus, Airway epithelium

## Abstract

Vegetable glycerin (VG) and propylene glycol (PG) serve as delivery vehicles for nicotine and flavorings in most e-cigarette (e-cig) liquids. Here, we investigated whether VG e-cig aerosols, in the absence of nicotine and flavors, impact parameters of mucociliary function in human volunteers, a large animal model (sheep), and air-liquid interface (ALI) cultures of primary human bronchial epithelial cells (HBECs). We found that VG-containing (VG or PG/VG), but not sole PG-containing, e-cig aerosols reduced the activity of nasal cystic fibrosis transmembrane conductance regulator (CFTR) in human volunteers who vaped for seven days. Markers of inflammation, including interleukin-6 (IL6), interleukin-8 (IL8) and matrix metalloproteinase-9 (MMP9) mRNAs, as well as MMP-9 activity and mucin 5AC (MUC5AC) expression levels, were also elevated in nasal samples from volunteers who vaped VG-containing e-liquids. In sheep, exposures to VG e-cig aerosols for five days increased mucus concentrations and MMP-9 activity in tracheal secretions and plasma levels of transforming growth factor-beta 1 (TGF-β1). *In vitro* exposure of HBECs to VG e-cig aerosols for five days decreased ciliary beating and increased mucus concentrations. VG e-cig aerosols also reduced CFTR function in HBECs, mechanistically by reducing membrane fluidity. Although VG e-cig aerosols did not increase MMP9 mRNA expression, expression levels of IL6, IL8, TGFB1, and MUC5AC mRNAs were significantly increased in HBECs after seven days of exposure. Thus, VG e-cig aerosols can potentially cause harm in the airway by inducing inflammation and ion channel dysfunction with consequent mucus hyperconcentration.

## Introduction

E-cigarettes (e-cigs) are generally thought to produce fewer toxins than combustible cigarettes leading to the notion that vaping e-cigs may be less harmful than smoking cigarettes. E-cig liquids (e-liquids) typically comprise high concentrations of nicotine, flavoring agents, and the delivery vehicles propylene glycol (PG) and vegetable glycerin (VG). While aerosols of e-liquids containing nicotine and PG/VG, both in the presence and absence of flavorings, can cause inflammation and harm in the airways (Wu et al., 2014; Garcia-Arcos et al., 2016; Hwang et al., 2016; Werley et al., 2016; Crotty Alexander et al., 2018; Ghosh et al., 2018; Chung et al., 2019), how these distinct constituents contribute to the damaging effects of e-cigs is less clear. Nicotine, on its own, can exert harmful effects on the airway epithelium (Maouche et al., 2013; Ahmad et al., 2019; Chung et al., 2019). Common flavoring agents, including cinnamaldehyde, diacetyl, and 2,3-pentanedione, have also been shown to disrupt mucociliary function (Clapp et al., 2019; Park et al., 2019). Moreover, many flavoring chemicals are known to exert toxic effects (Sherwood and Boitano, 2016; Muthumalage et al., 2017; Sassano et al., 2018). On the other hand, PG and VG are generally regarded as innocuous even though PG and VG comprise the bulk of the formulation of most e-liquids. However, evidence that aerosolization of PG and VG by e-cig devices produces toxicants suggests that PG and VG can impose their own unique harms to the airways.

E-cig aerosols of PG and/or VG, in the absence of nicotine and flavoring agents, have recently been shown to exert harmful effects in the airways both *in vitro* and *in vivo*. E-cig aerosols of PG/VG have been demonstrated to cause inflammation and disrupt glucose metabolism in exposed airway epithelial cells *in vitro* (Escobar et al., 2020; Woodall et al., 2020; Escobar et al., 2021). Furthermore, mice chronically exposed to e-cig aerosols were found to have disrupted lipid homeostasis in the lung, thereby impairing the immune response to infection (Madison et al., 2019). PG and VG in e-liquids have also been shown to be a major source of toxicants generated from e-cigs (Kosmider et al., 2014; Wang et al., 2017). Although these data provide initial evidence of the underlying dangers of PG and VG, the effects of these aerosols in human airways remain largely unknown.

Decreased mucociliary clearance (MCC) can be caused by dysfunction of ion channels that act to keep the airway properly hydrated. The cystic fibrosis transmembrane conductance regulator (CFTR) functions as an ion channel important for maintaining airway surface liquid (ASL) volumes for proper ciliary beating and mucus transport in the airway epithelium (Mall and Hartl, 2014). E-cig aerosols of nicotine and PG/VG, in the absence of flavorings, can inhibit CFTR function 8 h after exposure in primary human bronchial epithelial cells (HBECs) *in vitro* (Garcia-Arcos et al., 2016). VG only aerosols have also been shown to reduce CFTR activity in HBECs 10 min after exposure, possibly by the production of acrolein (Lin et al., 2019). How longer exposures to VG aerosols, in the absence of nicotine, affect CFTR function remains unclear. The impact of e-cig aerosols on CFTR conductance is particularly relevant given that CFTR dysfunction is associated with chronic bronchitis (Raju et al., 2016). Indeed, adolescent and young adult e-cig users have higher incidence of chronic bronchitis symptoms compared to those who never vaped (McConnell et al., 2017). Thus, elucidating the impact of realistic exposures of these delivery vehicles on ion channel and mucociliary function remains important to our understanding of the health effects of e-cigs.

In this study, we investigated the effects of week-long exposures to VG e-cig aerosols on airway inflammation and ion channel function. Both *in vitro* and *in vivo* systems were employed in this study: a small cohort of volunteers in a clinical study, a large animal model (sheep), and air-liquid interface (ALI) cultures of fully differentiated primary HBECs. Although the majority of e-cig users comprise former and current users of combustible cigarettes (Mayer et al., 2020), we chose to examine the airway effects of VG aerosols in naïve users with no previous history of smoking or vaping given the high prevalence of vaping among adolescents (Gentzke et al., 2022). Furthermore, we considered the possibility that elevated levels of inflammatory markers and mucin expression in the airways of combustible cigarette smokers (Reidel et al., 2018; Kim et al., 2021) could potentially mask the effects of VG aerosols at shorter exposures.

Thus, human volunteers who had never smoked nor vaped were initially recruited to assess effects of vaping PG/VG, PG or VG on ion transport and inflammatory responses in their upper airways before the study was halted due to the e-cigarette, or vaping, product use-associated lung injury (EVALI) outbreak (Krishnasamy et al., 2020). However, we found that a small sample of volunteers who vaped VG-containing e-liquids had reduced nasal CFTR activity after seven days, prompting us to further explore the contributions of VG e-cig aerosols to mucociliary dysfunction in sheep *in vivo* and HBECs *in vitro*. Sheep provide an ideal model system for *in vivo* studies of airway effects of e-cig aerosols because sheep airways resemble human airways regarding mucociliary function much more closely than murine airways and aerosol exposures can be carefully controlled in sheep (Abraham, 2008; Chung et al., 2019; Kim et al., 2020). Collectively, our data show that VG e-cig aerosols cause mucus hyperconcentration, airway inflammation, and reductions in CFTR activity, the latter by VG incorporation into the membrane.

## Methods

### E-cigarette device and e-liquids

The eVic Supreme™ (Joyetech, Shenzen, China) was used for its ability to store user’s vaping topography data as previously reported (Guerrero-Cignarella et al., 2018). Information was downloaded using the Joyetech myVapors^®^ software. The Delta 23 atomizer (Joyetech) was filled with either 100% PG (American E-liquid Store, Wauwatosa, WI, United States), 100% VG (American E-liquid Store), or 50%/50% v/v PG/VG mixture. The PG/VG blend was specifically mixed by Pace Engineering Concepts (Delafield, WI, United States) to assure quality control without additions to these nicotine- and flavoring-free e-liquids. The Delta 23 uses the C3 Atomizer Head which is a 3-coil device with a resistance of 1.4 Ω. Topography data, including the average daily voltage and wattage of the Delta 23 atomizer, is shown in Table 1. All e-liquids were custom made for research purposes and contained no nicotine or flavoring additives.

**TABLE 1:** Characteristics of recruited never-smoker, never-vaper participants.

Characteristic	Combined (*n* = 16)	PG group (*n* = 7)	VG group (*n* = 6)	PG/VG group (*n* = 3)
Age (yr) Mean Median Range	44.6 46.5 21–68	46.6 47 34–58	46.8 47.5 21–68	35.3 25 24–57
Female	15 (94%)	6 (86%)	6 (100%)	3 (100%)
Race/Ethnicity Caucasian Hispanic Other/Unknown	2 13 1	1 6 0	1 5 0	0 2 1
Puffs/day Mean Median Range	123.5 119.6 47.8–194.2	122.8 107.9 47.8–194.2	121 119.6 60.6–164.8	130.1 139.6 109.1–141.4
Duration/puff (s) Mean Median Range	3.3 2.7 1.2–7.0	3.7 3.3 1.2–7.0	3.6 4.0 1.8–5.1	2.3 2.3 2.1–2.6
Daily puff time (s) Mean Median Range	336.7 315 217.5–497.8	315.3 294.1 217.5–445	383.9 362.5 283.6–497.8	300.6 297 283.8–321.1
Average voltage Mean Median Range	3.4 3.3 3.0–4.7	3.5 3.5 3.0–4.5	3.5 3.3 3.0–4.7	3.2 3.3 3.0–3.3
Average wattage Mean Median Range	8.5 7.5 5.7–15.7	8.9 8.2 5.7–14.6	8.9 7.4 6.3–15.7	7.0 7.5 6.0–7.5

### Study population and design

The study protocol was reviewed and approved by the University of Miami Miller School of Medicine Institutional Review Board (IRB). The trial was registered in ClinicalTrials.gov (NCT02585791). Participants with no prior history of smoking or vaping were recruited for this study. All study volunteers were fully informed of study risks. After signing informed consent, basic demographic and social history information was collected. Baseline clinical measures, such as vital signs and pulmonary function test by spirometry (to exclude any previous pulmonary disease), were then collected. Volunteers were trained on how to use the eVic Supreme™ e-cig device, which captures topography data, and initially asked to vape at least 100 puffs daily for one week. They were randomized to vape e-liquids containing either 100% PG, 100% VG, or 50%/50% v/v PG/VG (American E-liquid Store; Pace Engineering Concepts). Only data from compliant participants were included in analyses. Compliance criteria were (i) exposure to an average of ≥ 40 puffs per day with ≥ 200 s daily puff time and (ii) completing their final visit for sample collections and measurements. A total of 23 volunteers were deemed eligible for the study, although 3 volunteers failed to complete the study because they failed to appear for follow-up visits. 7/7 (100%) participants in the PG group, 6/8 (75%) participants in the VG group, and 3/5 (60%) of participants in the PG/VG group were fully compliant and completed the study. Study participants received monetary compensation upon completion of each study visit.

### Nasal potential difference measurements

Nasal ion transport was assessed by measuring NPD according to Therapeutic Development Network standards of the Cystic Fibrosis Foundation (CFF) and as previously described (Rowe et al., 2011). NPD from volunteers was recorded at initial and final visits. A nasal catheter was inserted into the participant’s right nostril and a grounding electrode onto an abraded section of their forearm to measure a stable baseline potential difference between their inferior turbinate and skin. To start, a defined buffered Ringer’s solution (148 mM NaCl, 2.25 mM CaCl_2_, 4.05 mM KCl, 2.4 mM K_2_HPO_4_, 0.4 mM KH_2_PO_4_, 1.2 mM MgCl_2_) (Solution #1) was pumped at a rate of 5 ml min^−1^ until the recording stabilized. Next, Ringer’s solution containing amiloride (100 μM; Solution #2) was perfused. After the NPD recording stabilized once more, a chloride-free Ringer’s solution containing amiloride (“0 mM Chloride”; Solution #3) was perfused. Next, chloride-free Ringer’s solution containing amiloride and isoproterenol (10 μM; Solution #4) was perfused. The change caused by Solution #3 and #4 (chloride-free and isoproterenol) from Solution #2 was defined as nasal CFTR function in this study. NPD recordings of all 16 participants were performed at the initial and final visits, but only 7 participants completed the NPD measurements satisfactorily.

### Human nasal epithelial cell collection

HNECs were harvested from the nasal cavity by gentle nasal brushing three times using a 5.5-mm diameter nylon brush (Medical Packaging Corp, CA, United States) as previously described (Bengtson et al., 2020; Kim et al., 2021). The brush was swirled in a tube containing phosphate buffered normal saline to release the cells. HNECs were centrifuged, supernatant removed, and the remaining pellet was stored at −80°C until analysis.

### Nasal fluid collection

Nasal epithelial lining fluid (ELF) was collected using pre-cut strips of synthetic absorptive matrix (Leukosorb; Pall Corporation, Port Washington, NY, United States) as previously described (Kim et al., 2022b). Leukosorb was stored at −80°C before elution and sample analysis.

### Animal (sheep) study design

All procedures were approved by the Mount Sinai Medical Center Animal Research Committee. Although the response to VG aerosols may be influenced by the sex of the animal, only adult female sheep (ewes) were used in this study. Male sheep are naturally more aggressive and thus not amenable for long-term experimentation. Specifically, nasal intubation cannot be performed in males without the use of general anesthesia, hindering e-cig aerosol delivery during inspiration (see below) and potentially affecting measurements. For these studies, conscious ewes were nasally intubated, and their tracheal secretions and plasma were collected as previously described (Chung et al., 2019; Kim et al., 2020).

### E-cig aerosol exposure for sheep

Nasally intubated sheep were exposed to aerosols generated from an eVic Supreme™ (Joyetech, Shenzen, China) with e-liquid containing 100% VG. The Delta 23 atomizer was set to 3.2 V–3.6 V for a power setting of ∼7.0 W during aerosol collection. Aerosols were drawn into a 60-ml syringe and then delivered into the trachea only during inspiration, at a frequency of 20 breaths/min and a tidal volume of 500 ml, via the inspiration tubing of a piston respirator. Adult ewes were exposed to 80 puffs (40 puffs per session twice daily with ≥ 240 s daily puff time) of 100% VG aerosols for five consecutive days.

### Sheep mucus concentration (% mucus solids) measurements

Percent solids of mucus from sheep tracheal secretions was measured and calculated as previously described (Chung et al., 2019; Kim et al., 2020).

### MMP-9 activity measurements

MMP-9 activity was measured from human nasal ELF samples using a Human MMP-9 Activity Assay (#QZBMMP9H; QuickZyme Biosciences, South Holland, Netherlands). MMP-9 activity was measured from sheep tracheal secretions using a Human Active MMP-9 Fluorokine E kit (#F9M00; R&D Systems, Minneapolis, MN, United States) that was first validated for use with sheep.

### ELISA

Human MUC5AC ELISA Kit (#LS-F4836; LSBio, Seattle, WA, United States) was used for human nasal ELF samples. Nori^®^ Sheep TGF-β1 ELISA kit (#GR106127; Genorise, Berwyn, PA, United States), Nori^®^ Sheep IL-6 ELISA kit (#GR106329; Genorise), and Nori^®^ Sheep IL-8 ELISA kit (#GR106453; Genorise) were used for sheep plasma samples.

### Air-liquid interface cultures

Donor lungs were provided by organ procurement agencies, including the Life Alliance Organ Recovery Agency at the University of Miami (Miami, FL, United States), LifeCenter Northwest (Bellevue, WA, United States), the Nevada Donor Network (Las Vegas, NV, United States), and the Midwest Transplant Network (Westwood, KS, United States) after obtaining IRB-approved consent for research. HBECs were isolated from de-identified donor lungs of never-smoking individuals with no documented airways diseases but were rejected for transplant after informed consent. Donor lung demographics are listed in Supplementary Table S1. HBECs were cultured at the ALI according to published methods (Fulcher et al., 2005). HBECs were fully differentiated at the ALI for ≥ 4 weeks before e-cig aerosol exposures and as previously described (Garcia-Arcos et al., 2016; Chung et al., 2019).

### E-cig aerosol exposure for HBECs *in vitro*

The VC-1 exposure robot (Vitrocell, Heidelberg, Germany) was used for all e-cig aerosol exposures using slightly modified ISO parameters (Garcia-Arcos et al., 2016; Chung et al., 2019). A puff of e-cig aerosol was generated from 100% VG e-liquid using the Delta 23 atomizer set to 3.2 V–3.6 V for a power setting of ∼7.0 W during 3 s of aerosol collection. Batteries were fully charged before each exposure. ALI cultures were exposed to either filtered air or 50 puffs of aerosol (55 ml per puff, applied once every 20 s for 16.7 min). Each puff of e-cig aerosol was diluted with humidified air (relative humidity > 50%) at 0.25 L min^−1^. A constant vacuum was applied at 5 ml min^−1^ to generate sufficient flow for the e-cig aerosol to gently apply onto the ALI culture surface. HBECs were exposed to 50 puffs per session in the morning and evening (100 total puffs/day), for five or seven consecutive days. Basolateral media was changed every other day and the apical surface was unwashed during the experiments.

### Ciliary beat frequency measurements

CBF recordings were performed as previously described (Salathe and Bookman, 1999) on ALI cultures within a 3 mm radius from the center (free of influence by liquid meniscus) using a Basler acA645 camera (Basler, Ahrensburg, Germany) mounted on a Zeiss Axiovert running SAVA software (Sisson et al., 2003). CBF was recorded 24 h after last exposure.

### Mucus concentration (% solids) measurements of ALI cultures

The percentage of mucus solids on the apical surface of ALI cultures was measured according to published methods of wet and dry weights using a UMX2 microbalance (Mettler-Toledo, Columbus, OH, United States) (Anderson et al., 2015; Button et al., 2016). Percent mucus solids was measured 24 h after last exposure.

### LDH cytotoxicity assay

Cytotoxicity was assessed by measuring lactate dehydrogenase (LDH) in basolateral media from HBECs exposed to e-cig aerosols for five days using the CyQUANT™ LDH Cytotoxicity Assay kit (Thermo Fisher Scientific, Waltham, MA, United States).

### Ussing chamber

HBECs were mounted in Ussing chambers connected to a VCC MC6 or MC8 voltage clamp unit (Physiologic Instruments, San Diego, CA, United States). CFTR function was measured as the change in short-circuit current (I_*SC*_) caused by CFTR inhibition with 10 µM CFTR_inh_-172 (#C2992; MilliporeSigma, Burlington, MA, United States) after CFTR stimulation with 10 µM forskolin (#F3197; MilliporeSigma) in the presence of 10 µM amiloride (#A7410; MilliporeSigma) under a basolateral-to-apical chloride gradient as previously described (Garcia-Arcos et al., 2016; Sailland et al., 2017). Calcium-activated chloride channels (CaCC) conductance was measured as the change in I_*SC*_ caused by UTP (10 µM) stimulation following CFTR_inh_-172.

### Membrane fluidity measurements

Relative membrane fluidity was assessed using merocyanin 540 (MC540; Chemodex, St. Gallen, Switzerland) as previously described (Ghosh et al., 2018). Briefly, MC540 has been shown to partition into lipid layers and to change its emission properties based on membrane fluidity. HBECs were loaded with MC540 by incubating 300 µl of 100 µM MC540 (in 1x PBS, pH 7.4, 37°C) on the apical surface for 30 min. The apical surface was then washed twice with 1x PBS, pH 7.4, and cultures were incubated overnight prior to e-cig aerosol exposure. MC540 emissions were recorded from 550 nm to 700 nm in 10 nm increments using a plate-reader (Ex: 540 nm) before and after e-cig aerosol exposure. All MC540 emissions were normalized to 550 nm values and an area-under-curve (AUC) was calculated for each sample before and after e-cig aerosol (or control exposure). The difference in AUC for MC540 emissions (ΔAUC_MC540_) was used to assess changes in membrane fluidity. To directly elicit changes in membrane fluidity, cholesterol oxidase (COase; MP Biomedical, Irvine, CA, United States) and cholesterol esterase (CEase; MP Biomedical) were used as previously described (Abu-Arish et al., 2015). HBECs mounted onto Ussing chambers were apically exposed to COase (1 U/ml), CEase (0.2 U/ml), or buffer control for 30 min prior to measuring ion transport in Ussing chambers.

### Quantitative real-time PCR

RNA was isolated from HNECs and HBECs using the E. Z.N.A. Total RNA Kit (OMEGA Bio-tek, Norcross, GA, United States) and cDNA was made using the iScript cDNA synthesis kit (Bio-Rad, Hercules, CA, United States). qPCR was performed for each sample using a TaqMan Universal Master Mix (Thermo Fisher Scientific) mixed with TaqMan primers for target genes which include *IL1B* (Hs01555410_m1), *IL6* (hs00985639_m1), *IL8* (hs00174103_m1), *MMP2* (Hs01548727_m1), *MMP9* (Hs00234579_m1), and *TGFB1* (hs01365601_M1). *GAPDH* (Hs99999905_m1) was used as endogenous control. Expression data was generated by ΔΔC_T_ method using the threshold cycle (C_T_) value of target gene and *GAPDH*.

### Statistics

Presented data are mean ± SEM and were analyzed by PRISM V9 software (GraphPad, San Diego, CA, United States). Data were considered significant if p < 0.05 for compared means with respective parametric or non-parametric tests. Normality was assessed by Shapiro-Wilk. Statistical differences were queried using group comparison tests deemed appropriate for collected data. Multiple-testing correction was not performed. Additional information on statistical analyses is described in the figure legends.

## Results

### Week-long vaping with VG-containing e-liquids reduces nasal CFTR activity and increases the expression of inflammation markers in human volunteers

**FIGURE 1:**
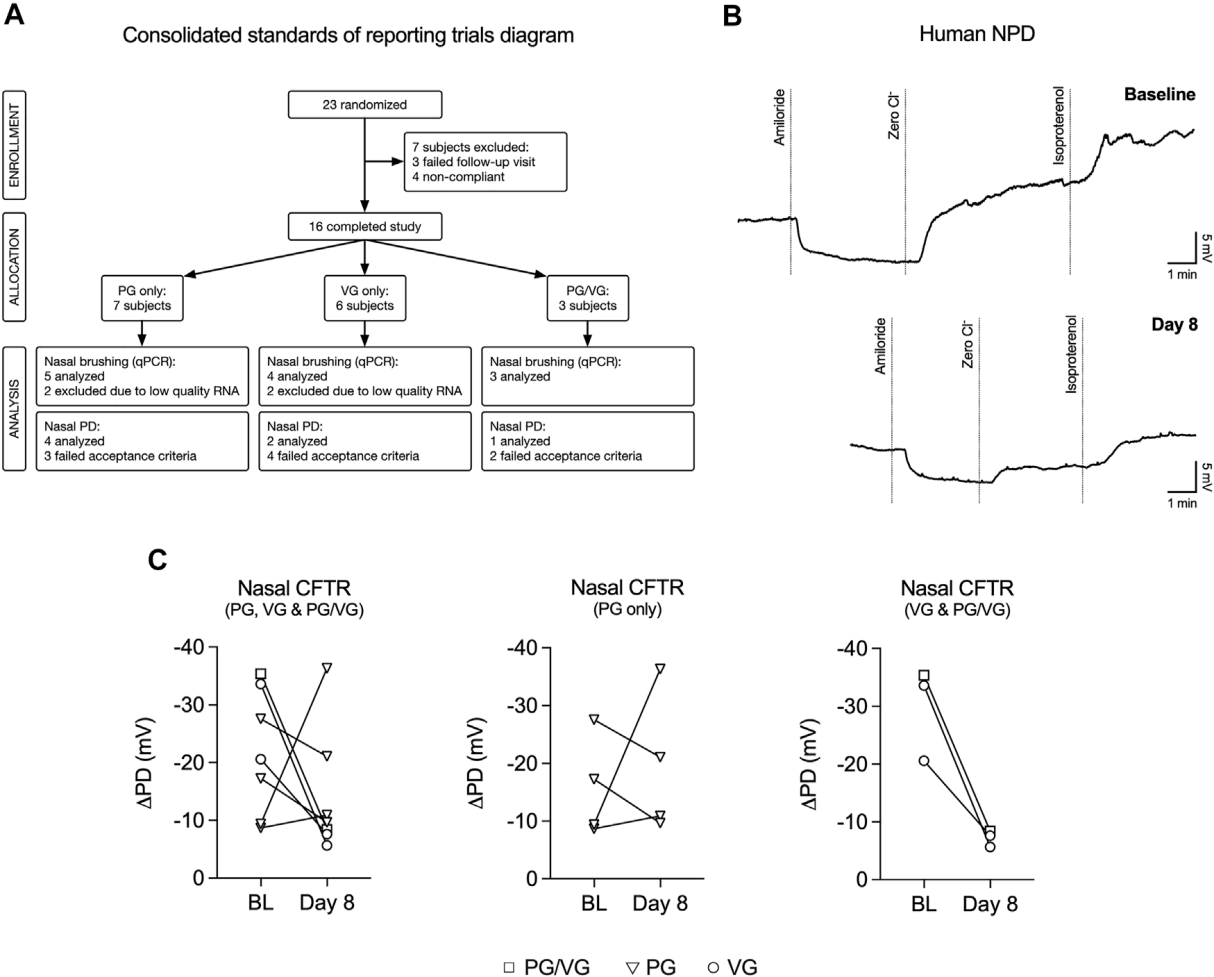
VG and PG/VG e-cig aerosols impair nasal CFTR function *in vivo*. **(A)** Consolidated Standards of Reporting Trials diagram demonstrating the flow of participants and their samples used in this study. Compliance was defined as initiating at least 40 puffs per day with ≥ 200 s daily puff time. Nasal potential difference (NPD) recordings and nasal brushings were collected at baseline and Day 8. **(B)** Example NPD recordings from a volunteer who vaped VG e-liquid performed at initial (baseline) and final (Day 8) visits. **(C)** Before-after plots of nasal CFTR function (measured as change following “Zero Cl^−^” and “Isoproterenol” treatments) from all volunteers at baseline (BL) and at Day 8 after one week of vaping. *n* = 7. The reduction in nasal CFTR activity is seen in all volunteers who inhaled aerosol generated from e-liquids containing VG (PG/VG and VG only; *n* = 3).

We conducted a study to investigate the effects of e-cig aerosols of PG and/or VG on the upper airways of never-smoking, never-vaping human volunteers. A total of 23 volunteers who had no previous history of smoking or e-cig use participated in the study before recruitment was halted prematurely due to updated National Institutes of Health (NIH) guidelines for e-cig-related trials due to the EVALI outbreak (Krishnasamy et al., 2020). The study flow chart is shown in Figure 1A and participant characteristics are reported in Table 1. Volunteers were trained on how to use the eVic™ Supreme device, which is capable of recording topography data. This allowed for volunteers to vape *ad libitum* for seven days in their normal environment. Volunteers were randomly assigned to vape e-liquids containing either 100% PG, 100% VG, or 50%/50% v/v PG/VG for one week. Since a previous study by us demonstrated that the average duration per puff and total daily puff time are good indicators of e-cig device use (Guerrero-Cignarella et al., 2018), compliance was defined as initiating at least 40 puffs per day with ≥ 200 s daily puff time.

CFTR responses were assessed by nasal potential difference (NPD) measurements (Knowles et al., 1995), which were performed at baseline and on day 8 (Figure 1B). Only seven participants completed both NPD tests satisfactorily. The voltage change response to chloride-free and isoproterenol perfusion (measure of CFTR activity) in volunteers from all groups (PG, VG, and PG/VG) trended towards a decrease after seven days of vaping (Figure 1C). However, when stratified into groups, CFTR conductance was found to be reduced in all volunteers who inhaled e-cig aerosols containing VG (VG only and PG/VG; Figure 1C).

**FIGURE 2:**
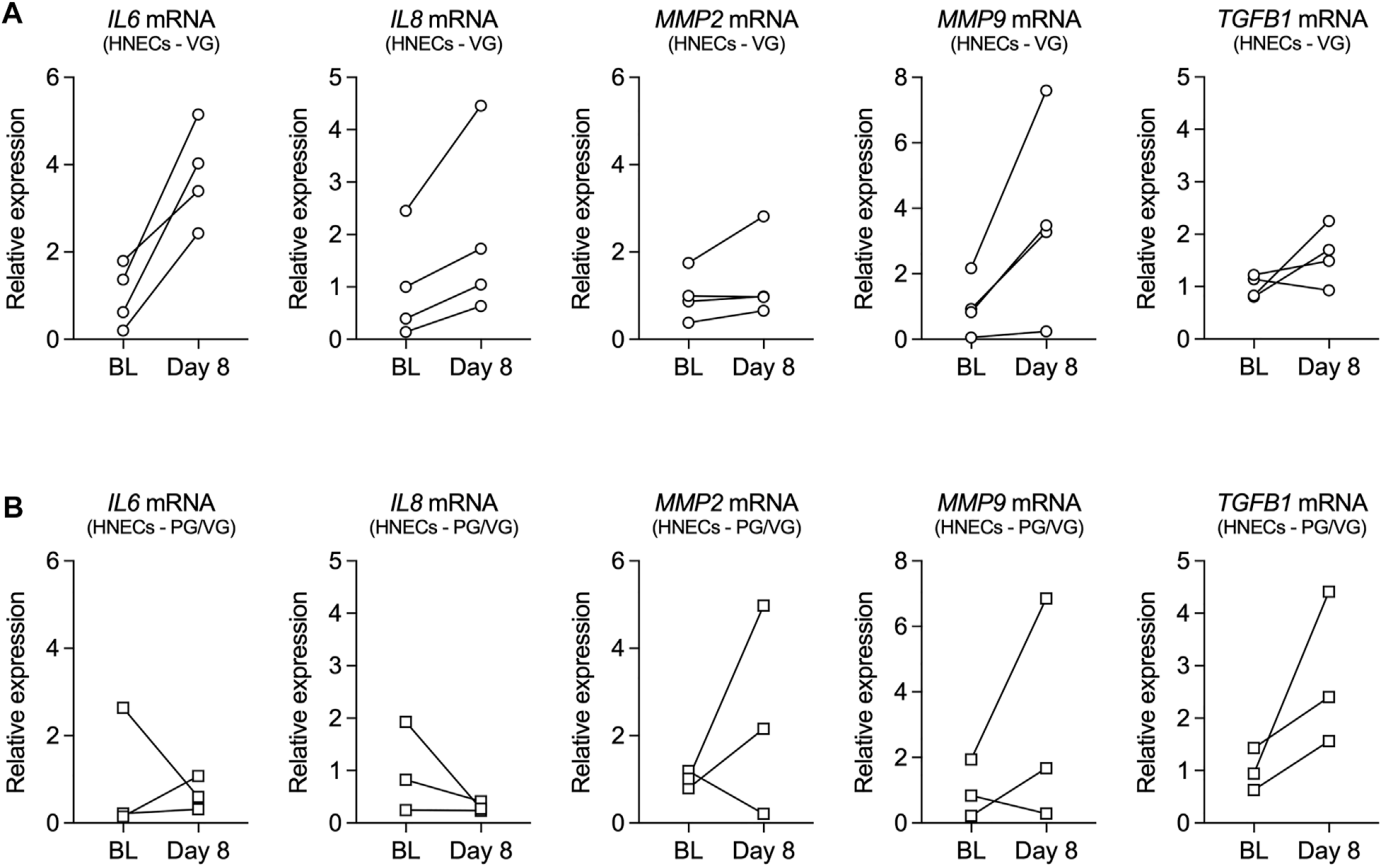
Inflammatory markers in nasal cells from volunteers who vaped VG and PG/VG e-liquids. **(A,B)** Before-after plots of mRNA expressions of *IL6*, *IL8, MMP2, MMP9,* and *TGFB1* measured in HNECs at baseline (BL) and day 8 from volunteers who vaped VG **(A)** or PG/VG **(B)**. *n* = 4 for VG, *n* = 3 for PG/VG.

mRNA expression of inflammatory mediators, including *interleukin-6* (*IL6*), *interleukin-8* (*IL8*), *matrix metalloproteinase-2* (*MMP2*), *matrix metalloproteinase-9* (*MMP9*), and *transforming growth factor beta 1* (*TGFB1*), was further measured from human nasal epithelial cells (HNECs) collected at baseline and on day 8 from volunteers who vaped PG/VG or VG. Although the small sample size did not allow for statistical comparisons, expression levels of *IL6*, *IL8*, and *MMP9* mRNAs were increased in HNECs from all volunteers who vaped VG aerosols after one week (Figure 2A). Expression levels of *MMP2* and *TGFB1* mRNAs also trended toward an increase in the VG group (Figure 2A). On the other hand, the effect of vaping PG/VG aerosols on the expression of inflammatory markers in HNECs was mixed (Figure 2B).

**FIGURE 3:**
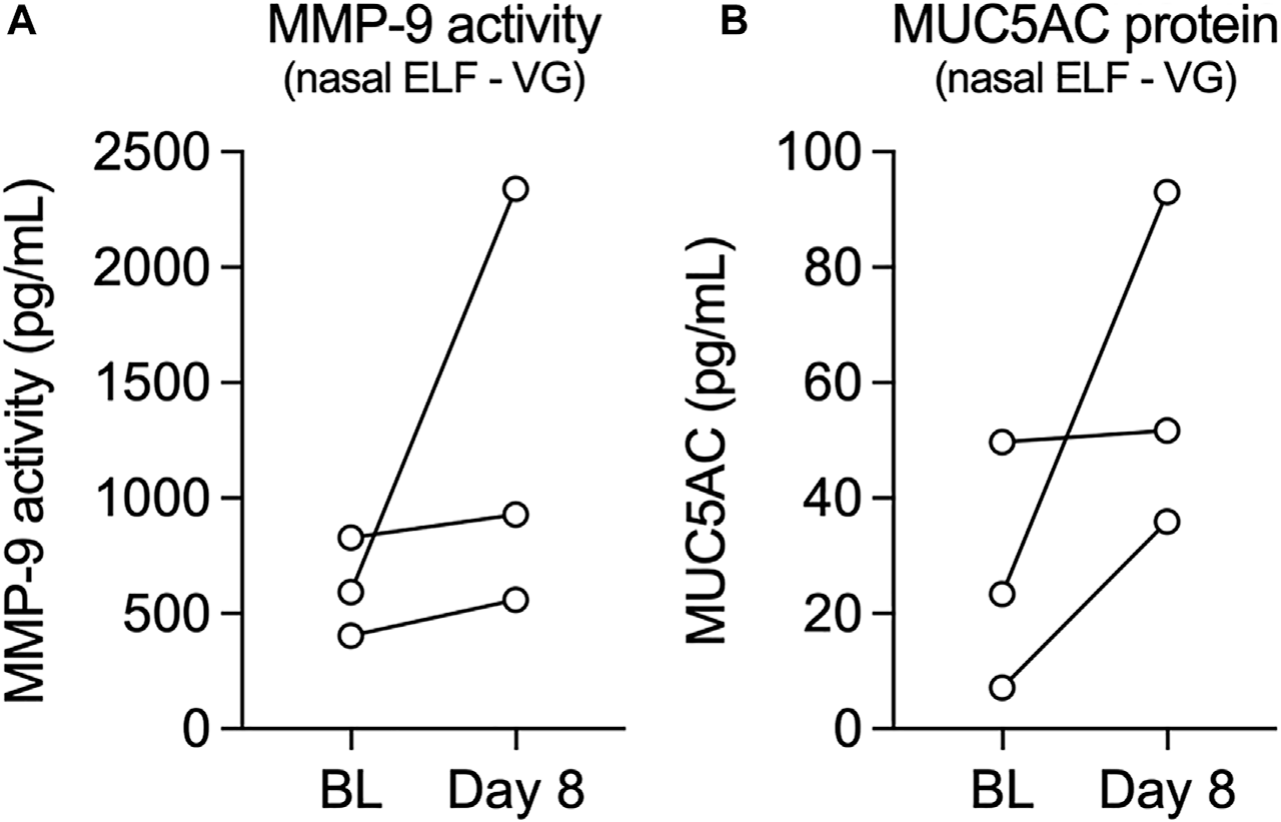
Levels of MMP-9 activity and MUC5AC protein in nasal epithelial lining fluid (ELF) from volunteers who vaped VG e-liquid. **(A,B)** Before-after plots of levels of MMP-9 activity **(A)** and MUC5AC protein **(B)** measured from nasal ELF at baseline (BL) and day 8 from volunteers who vaped VG. *n* = 3.

We next measured activity levels of MMP-9 and expression levels of mucin 5AC (MUC5AC) from nasal epithelial lining fluid (ELF) of volunteers who vaped VG e-cig aerosols. Leukosorb collections of nasal ELF provide a reliable and non-invasive sampling of the nasal mucosa for the analysis of inflammatory biomarkers (Rebuli et al., 2017). MMP-9 activity levels increased after one week in all volunteers who vaped VG aerosols (Figure 3A), consistent with the increase in *MMP9* mRNA expression observed in HNECs. Levels of MUC5AC in nasal ELF also increased after one week in all volunteers in the VG group (Figure 3B).

### VG e-cig aerosols increase mucus concentrations and MMP-9 activity in sheep airways and plasma levels of TGF-β1 *in vivo*.

**FIGURE 4:**
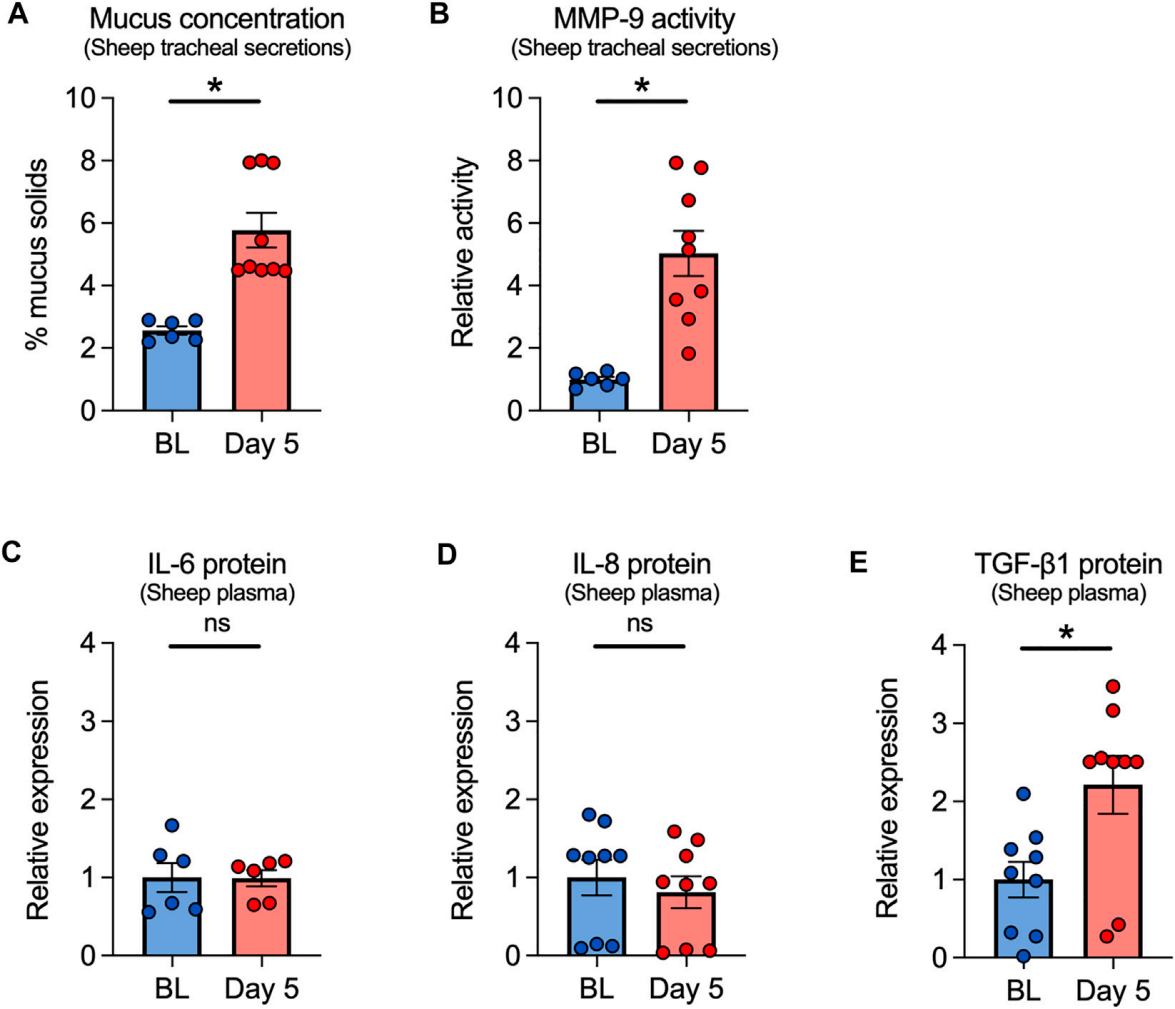
Effects of VG e-cig aerosols in a large animal model (sheep). **(A)** Five-day exposure of sheep to 100% VG e-cig aerosols causes a significant increase in mucus concentrations (measured as % mucus solids from tracheal secretions) after five days. *n* = 6 from 2 sheep for baseline, *n* = 9 from 3 sheep for Day 5. **(B)** Five-day exposure of sheep to VG aerosols causes a significant increase in MMP-9 activity measured from tracheal secretions. *n* = 6 from 2 sheep for baseline, *n* = 9 from 3 sheep for Day 5. **(C–E)** Five-day exposure of sheep to VG e-cig aerosols does not cause a significant change in plasma levels of IL-6 **(C)** or IL-8 **(D)** proteins but causes a significant increase in the expression of TGF-β1 protein **(E)** in plasma. *n* = 6 from 2 sheep **(C)** or *n* = 9 from 3 sheep **(D,E)**. Statistics: Data are presented as mean ± SEM. *p < 0.05, ns = not significant. Data were analyzed using a mixed-effects model.

Since ovine and human airways bear close functional resemblance (Abraham, 2008; Kim et al., 2020), we used our established sheep e-cig exposure model to further investigate the effects of VG e-cig aerosols on the airways (Chung et al., 2019). Although most e-liquids contain both PG and VG, we focused solely on VG aerosols to avoid any confounding effects of PG. For these experiments, adult ewes were exposed to 80 puffs (40 puffs per session twice daily with ≥ 240 s daily puff time) of 100% VG aerosols generated by the eVic™ Supreme for five consecutive days. Tracheal secretions and blood were collected at baseline and on day 5 after the last exposure. Mucus concentrations measured from tracheal secretions were significantly increased in VG-exposed sheep after five days (Figure 4A). Because e-cigs can stimulate the release of proteases, including MMP-9 (Higham et al., 2016; Ghosh et al., 2019), we measured the activity of MMP-9 in tracheal secretions. MMP-9 activity was also significantly increased in tracheal secretions from sheep exposed to VG e-cig aerosols (Figure 4B).

We further measured the expression of inflammatory mediators, including IL-6, IL-8, and TGF-β1 in the plasma of VG-exposed sheep. While plasma levels of IL-6 or IL-8 proteins were unchanged after 5 days of exposure (Figures 4C,D), plasma levels of TGF-β1 significantly increased in VG-exposed sheep (Figure 4E).

### VG e-cig aerosols increase mucus concentrations and decrease ciliary beating in HBECs *in vitro*

**FIGURE 5:**
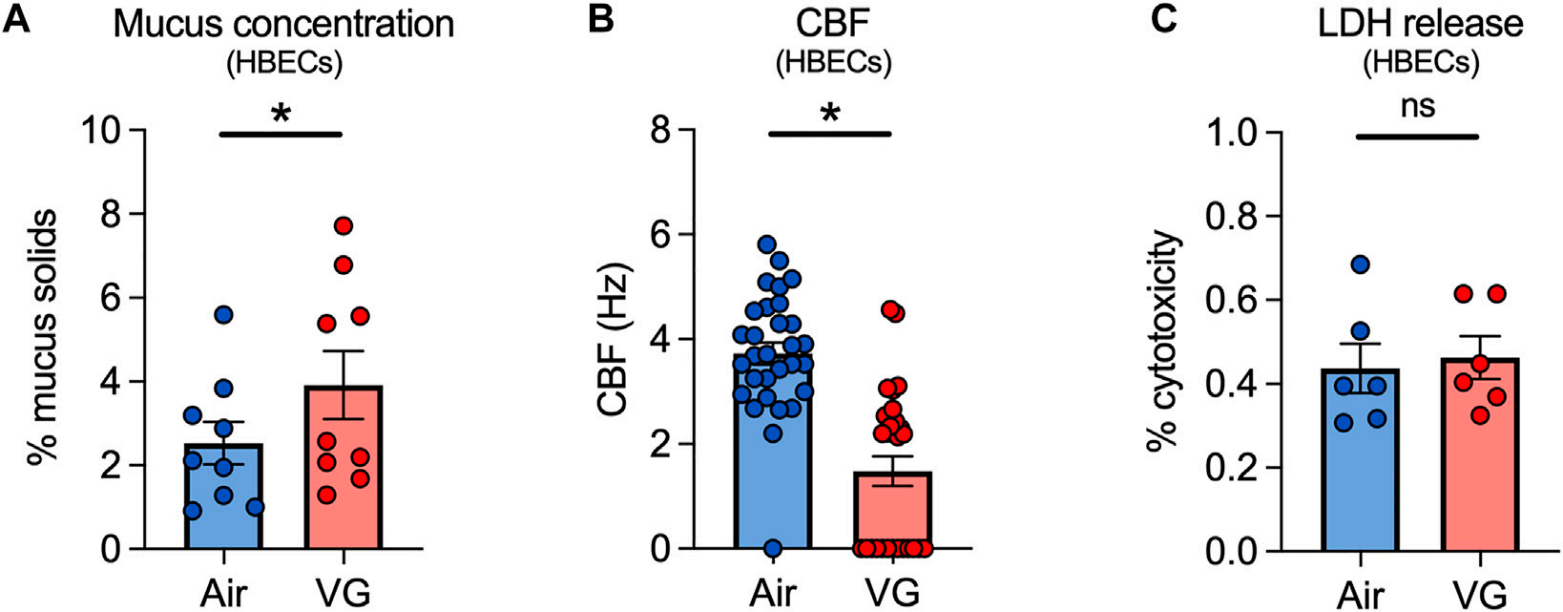
VG e-cig aerosols induce mucociliary dysfunction in primary HBECs *in vitro*. **(A)** Mucus concentrations are significantly increased in HBECs exposed to VG e-cig aerosols after five days compared to air-exposed controls. *n* = 6 from 5 lungs. **(B)** Ciliary beat frequency (CBF) of HBECs exposed to VG e-cig aerosols is significantly reduced after five days compared to air-exposed controls. *n* ≥ 23 from 5 lungs. **(C)** Five-day exposure of HBECs to VG e-cig aerosols does not affect cytotoxicity as assessed by lactate dehydrogenase (LDH) release into basolateral media. *n* = 6 lungs. Statistics: Data are presented as mean ± SEM. *p < 0.05, ns = not significant. Data were analyzed by two-tailed *t*-test **(A,C)** or mixed-effects model **(B)** after assessing normality by Shapiro-Wilk.

We next exposed primary HBECs to VG e-cig aerosols to investigate the direct effects of VG on the airway epithelium *in vitro*. HBECs from non-smoker donors were exposed to 100% VG e-cig aerosols for five days to mirror the five-day VG exposure in our sheep model. The VC-1 exposure robot was used to expose ALI cultures to 100 puffs per day (50 puffs per session twice daily) with a 3 s duration per puff for a total daily puff time of 300 s. VG-exposed HBECs showed a significant increase in mucus concentrations after five days compared to air-exposed controls (Figure 5A). CBF was also significantly decreased in HBECs after five days of exposure to VG e-cig aerosols (Figure 5B). Importantly, the reduction in CBF was unlikely due to cytotoxicity caused by VG aerosols (Figure 5C).

### VG e-cig aerosols cause CFTR dysfunction by altering membrane fluidity of HBECs *in vitro*.

**FIGURE 6:**
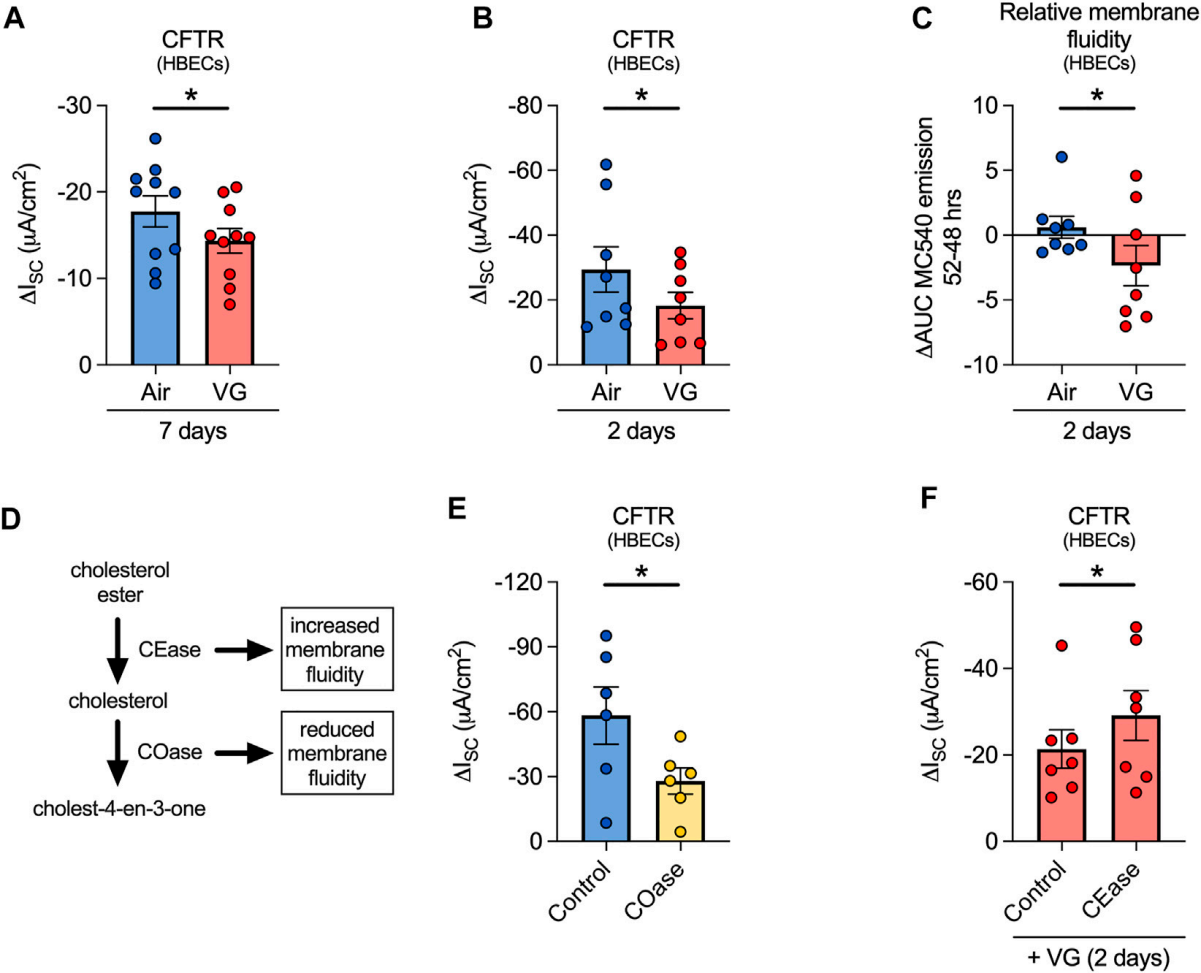
VG aerosols impair CFTR function in primary HBECs *in vitro* by reducing membrane fluidity. **(A)** VG only aerosols cause a significant reduction in CFTR function compared to air-exposed HBECs after seven days. *n* = 10 lungs. **(B)** HBECs exposed to VG aerosols for two days have significantly reduced CFTR function compared to air only controls. *n* = 8 lungs. **(C)** VG exposure reduces total MC540 emissions, indicative of reduced membrane fluidity, while air only controls do not cause a change in MC540 emissions. *n* = 8 lungs. **(D)** Schematic illustrating the activities of cholesterol oxidase and cholesterol esterase in cholesterol metabolism pathway. **(E)** To reduce membrane fluidity, HBECs were acutely treated with cholesterol oxidase (COase, 1 U/ml, 30 min). COase-treated HBECs have significantly reduced CFTR function compared to buffer only controls. *n* = 6 from 4 lungs. **(F)** To increase membrane fluidity, VG-exposed HBECs were treated with CEase (0.2 U/ml, 30 min). CEase significantly improves CFTR function. *n* = 7 lungs. Statistics: Data are presented as mean ± SEM. *p < 0.05, ns = not significant. Data were analyzed by two-tailed paired *t*-test **(A,B)**, one-tailed Wilcoxon test **(C)**, or one-tailed paired *t*-test **(E,F)** depending on normality assessment by Shapiro-Wilk.

We then set out to determine whether decrements in nasal CFTR function in volunteers who vaped VG-containing e-liquids could be recapitulated in primary HBECs *in vitro*. For these experiments, ALI cultures were exposed to e-cig aerosols of VG for seven days to mirror the seven-day exposure in our human volunteers. VG e-cig aerosols caused a significant reduction in CFTR function compared to air-exposed controls (Figure 6A). VG aerosols did not affect expression levels of *CFTR* mRNA (Supplementary Figure S1), suggesting that reduced CFTR function was not caused by decreased *CFTR* mRNA expression.

While aerosols of VG have been previously shown to affect CFTR conductance (Lin et al., 2019), further delineation of the mechanism was needed. E-cig liquids have been reported to alter membrane fluidity (Ghosh et al., 2018), but whether this can affect CFTR function has not been directly tested. Thus, we investigated whether aerosols containing VG might affect CFTR function via dynamic changes in membrane fluidity, assessed by monitoring fluorescence of merocyanine 540 (MC540), a lipophilic dye with increased affinity for fluid phased lipid bilayers (Williamson et al., 1983). Two-day exposures of HBECs to aerosols of 100% VG were sufficient to cause a significant decrease in CFTR function (Figure 6B) with a simultaneous, significant reduction in MC540 fluorescence (Figure 6C), indicating a decrease in membrane fluidity. Compared to seven-day exposures, two-day VG exposures caused a greater decline in CFTR activity, suggestive of an adaptive response of HBECs to prolonged exposures to VG aerosols.

We next determined whether decreasing membrane fluidity is sufficient to impair CFTR function. The fluidity of the membrane is influenced by cholesterol content, which itself can be altered by the actions of cholesterol oxidase (COase) and cholesterol esterase (CEase) (Figure 6D). ALI cultures treated apically for 30 min with COase, which decreases membrane fluidity by removing cholesterol, showed a reduction in CFTR function similar to VG aerosol exposure (Figure 6E). On the other hand, the activity of another chloride channel, calcium-activated chloride channels (CaCC), was not significantly affected by COase treatment (Supplementary Figure S1). We then tested whether increasing membrane fluidity can reverse the negative effects of VG aerosols on CFTR conductance. VG-exposed HBECs treated apically for 30 min with CEase, which increases membrane fluidity by catalyzing the hydrolysis of cholesterol esters into cholesterol, showed significantly improved CFTR function compared to controls (Figure 6F). CEase did not change CaCC conductance in VG-exposed cultures (Supplementary Figure S1). These data suggest that aerosols of VG preferentially affect CFTR activity by reducing membrane fluidity, possibly through VG incorporation into the membrane.

### VG e-cig aerosols increase the expression of inflammatory markers in HBECs *in vitro*.

**FIGURE 7:**
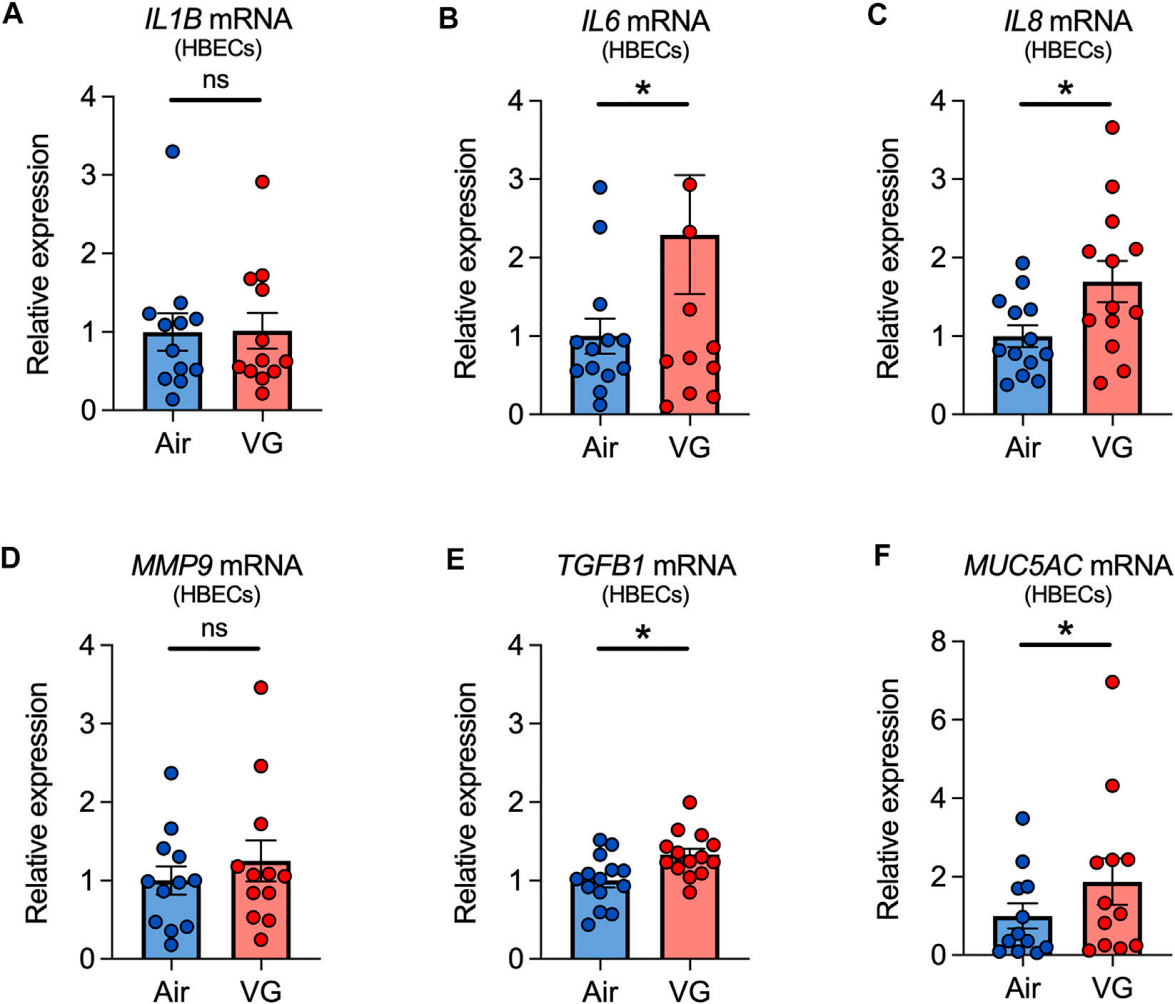
Inflammatory markers in primary HBECs exposed to VG e-cig aerosols. **(A–E)** VG aerosol exposure significantly increases *IL6* **(B)**, *IL8* **(C)**, and *TGFB1* **(E)**, but not *IL1B* **(A)** and *MMP9* **(D)**, mRNA expressions in HBECs after seven days. **(F)** Seven-day exposure of HBECs to VG aerosols causes a significant increase in *MUC5AC* mRNA expression. *n* ≥ 12 lungs. Data are shown as relative to *GAPDH* and air control. Statistics: Data are presented as mean ± SEM. *p < 0.05, ns = not significant. Data were analyzed by two-tailed Wilcoxon test or two-tailed paired *t*-test depending on normality assessment by Shapiro-Wilk.

Finally, we tested whether VG e-cig aerosols can elicit an inflammatory response. HBECs exposed to aerosols of VG showed significant increases in the expressions of *IL6*, *IL8*, and *TGFB1* mRNAs after seven days (Figure 7). However, expression of *interleukin-1 beta* (*IL1B*) and *MMP9* mRNAs was statistically unchanged after seven days of VG exposure (Figures 7A,D). VG aerosols caused a significant increase in the expression of *MUC5AC* in HBECs after seven days (Figure 7F).

## Discussion

We investigated the effects of nicotine- and flavoring-free e-cig aerosols on the airway, focusing on ion transport mechanisms, mucus production, and inflammation. Surprisingly, we found that nasal CFTR function of human volunteers who had never smoked nor vaped was reduced after only one week of vaping with VG-containing e-liquids (VG and PG/VG). The change in CFTR function was accompanied by elevated levels of inflammatory biomarkers, including *IL6*, *IL8*, and *MMP9* mRNAs and MMP-9 activity, in the upper airways of volunteers who vaped VG. These findings were consistent with *in vitro* experiments whereby seven-day exposures of never-smoker HBECs to VG e-cig aerosols similarly reduced CFTR channel function*.* Although CFTR dysfunction caused by acute exposure to e-cig aerosols of PG/VG is seemingly nicotine-dependent (Garcia-Arcos et al., 2016), our data here show that even two-day exposures of HBECs to VG only aerosols can significantly reduce CFTR activity. Mechanistically, VG aerosols reduced membrane fluidity and acutely increasing membrane fluidity restored CFTR function. In sheep, VG aerosols also increased mucus concentrations and MMP-9 activity in tracheal secretions. VG aerosols further elevated plasma TGF-β1 levels in sheep after only five days of exposure. Collectively, these data demonstrate that inhaling aerosols generated with VG-containing vehicles may be harmful.

Given these findings, the question is whether the observed changes are biologically meaningful. Although VG e-cig aerosols caused relatively small decrements in CFTR activity in HBECs *in vitro*, we observed significant changes in CBF and mucus concentrations that have been associated with mucociliary dysfunction (Anderson et al., 2015). Similar aerosol exposure conditions in sheep also produced increased mucus concentrations in tracheal secretions, though ion transport could not be directly tested in these animals. However, a small number of human volunteers who vaped VG-containing e-liquids for seven days also showed remarkable decreases in nasal CFTR activity similar to changes observed in the NPD response of volunteers to cigarette smoke (Clunes et al., 2012). Nasal CFTR activity in smokers with or without chronic obstructive pulmonary disease (COPD) is significantly impaired and associated with symptoms of chronic bronchitis (Sloane et al., 2012; Dransfield et al., 2013), suggesting observed changes in response to VG-containing aerosols could potentially lead to reduced MCC in e-cig users. Whether VG aerosols can further impair nasal CFTR activity in smokers (former and current), who comprise the majority of e-cig users, will be of considerable interest.

For our clinical study, we tasked fully informed volunteers with no previous history of smoking or e-cig use to vape PG, VG, or PG/VG e-liquids for one week to test whether nicotine- and flavoring-free e-cig aerosols have effects on the upper airways. Total number of recruited volunteers and completed NPD recordings (before and after) were a limitation of this study. Furthermore, most of our study population comprised female volunteers. Gender comparisons are important given that e-cig aerosols of PG, without nicotine, can influence inflammation in a sex-dependent manner in mice (Wang et al., 2019; Wang et al., 2020). However, the recruitment of additional male volunteers who were never-smokers and never-vapers was halted following the EVALI outbreak of 2019, making these comparisons impossible in this study. Despite these limitations, we observed that seven days of e-cig aerosol exposure trended towards reduced nasal CFTR function in our volunteers as a whole. Notably, those who inhaled VG only or PG/VG aerosols all showed reduced nasal CFTR function, consistent with CFTR responses to VG e-cig aerosols *in vitro*. Recent epidemiological studies reported that adolescent e-cig users experience chronic bronchitis symptoms (McConnell et al., 2017) and e-cig use increased risk for developing chronic bronchitis and other respiratory diseases (Bhatta and Glantz, 2020). Therefore, it is possible that exposure to aerosols of VG could contribute to increased risk for developing symptoms of chronic bronchitis.

Although VG aerosols have been shown to have harmful effects, little is known of how they specifically impact mucociliary function. Heating VG can generate acrolein, a major toxicant found in cigarette smoke that is highly reactive and reduces CFTR channel function by disrupting CFTR gating (Raju et al., 2017; Lin et al., 2019). However, our data here also support an alternate model whereby disruption of membrane fluidity by absorption of VG (Ghosh et al., 2018) causes CFTR dysfunction. We observed that aerosols of VG concomitantly reduced CFTR function while decreasing membrane fluidity. Acutely decreasing membrane fluidity with COase also reduced CFTR function. In contrast, acutely increasing membrane fluidity with CEase restored CFTR conductance in VG-exposed cultures. On the other hand, VG exposure and changes in membrane fluidity did not significantly affect the conductance of a different chloride channel, namely CaCC. Although it is unclear how changes in membrane fluidity specifically affect CFTR activity, there is an emerging link between cholesterol and the regulation of CFTR function (Cottrill et al., 2020). For example, surface expression of CFTR in cholesterol-rich lipid rafts can regulate its stability, which can be perturbed by changing the cholesterol content in the membrane (Abu-Arish et al., 2015; Abu-Arish et al., 2019). A recent study further showed that depletion of membrane cholesterol significantly reduces the ability of forskolin to activate CFTR (Cui et al., 2021), consistent with our results shown here.

We also tested for markers of inflammation following VG and PG/VG aerosol exposures. Despite the low sample size of our volunteer study, all individuals who vaped VG only e-liquids showed increases in the expressions of *IL6*, *IL8*, and *MMP9* mRNAs, as well as levels of MMP-9 activity and MUC5AC expression. The increase in MMP-9 expression and activity is particularly notable given known roles for MMP-9 in the pathogenesis of chronic airway diseases (Churg et al., 2012). Moreover, we recently showed that airway cells from smokers had significantly increased *MMP9* expression compared to non-smokers (Kim et al., 2021). We further found a significant increase in MMP-9 activity levels in the upper airways of smokers who switched to nicotine-containing e-cigs after 12 weeks (Kim et al., 2022a). Increased MUC5AC levels are also indicative of airway inflammation and are associated with muco-obstructive diseases (Radicioni et al., 2021). Although statistical comparisons were not possible because of small sample size, the increase in expression of these markers across all volunteers in the VG group demonstrates the potential pro-inflammatory consequences of vaping VG aerosols.

Overall, the data from our human studies, including increases in the mRNA expressions of *IL6* and *IL8*, and MUC5AC protein, are largely congruent with our *in vitro* data*.* Similar findings were reported by us and others under more acute settings (Garcia-Arcos et al., 2016; Singh et al., 2019), including recent studies showing that nicotine-free e-cig aerosols induced IL-6 secretion *in vitro* after 24 h (Escobar et al., 2020; Gellatly et al., 2020). Surprisingly, VG only aerosols also increased plasma levels of TGF-β1 in our sheep model *in vivo*. Although other inflammation markers remained unchanged (e.g., IL-6 and IL-8), systemic markers of inflammation have only been reported thus far after chronic exposure of nicotine-containing e-cig aerosols in mice (Crotty Alexander et al., 2018) and in e-cig users who vaped nicotine-containing products (Singh et al., 2019).

Although e-liquids and pod-based e-cigs contain both PG and VG, and in many cases higher ratios of PG, it remains important to delineate the individual contributions of these respective vehicles. PG/VG ratios impact the production of toxicants (Conklin et al., 2018; Li et al., 2021) and discrepancies regarding the effects of PG/VG aerosols in the lung are likely due to different exposure paradigms, including atomizer settings and duration of exposures (Phillips et al., 2017). This study represents one of the first attempts to systematically investigate the effects of VG only aerosols in the airway, particularly in human study participants. Despite the short duration of VG aerosol exposures (≤ 7 days), we observed changes in MMP-9 activity levels in both human and sheep airways. Although levels of MMP-2 and MMP-9 proteins and activities were found to be elevated in bronchoalveolar lavage fluid from e-cig users, these users vaped nicotine-containing e-liquids for at least six months (Ghosh et al., 2019). Interestingly, PG only aerosols did not affect MMP-9 expression in mice lungs after acute (3 days) exposure but caused a significant decrease after sub-chronic (30 days) exposure (Wang et al., 2019; Wang et al., 2020). Thus, it will be important to address the consequences of longer VG aerosol exposures in future studies.

In conclusion, this study demonstrates that aerosols from VG-containing e-cig liquid vehicles negatively impact airway ion transport, mucus concentrations, and inflammation in naïve airway epithelial cells, in sheep trachea, and in never-smoking and never-vaping volunteers. This occurred in the absence of nicotine and flavorings. There were many similar outcomes across all three model systems, such as impact on inflammation and mucus production, and some differences despite attempts to maintain similar exposure conditions (number of puffs, duration of exposure, identical e-cig liquids, and e-cig device). Overall, these findings demonstrate that VG e-cig aerosols, even in the absence of nicotine or flavors, have possibly harmful effects on the airways.

## Data Availability

The raw data supporting the conclusions of this article will be made available by the authors, without undue reservation.
