# Supplementary material for: Vegetable glycerin e-cigarette aerosols cause airway inflammation and ion channel dysfunction
Source: Front Pharmacol. 2022 Sep 26;13:1012723. doi: 10.3389/fphar.2022.1012723 (PMC9549247; doi:10.3389/fphar.2022.1012723)
Supplement: Supplementary file 1 [file DataSheet1.pdf]

Supplementary Figure S1

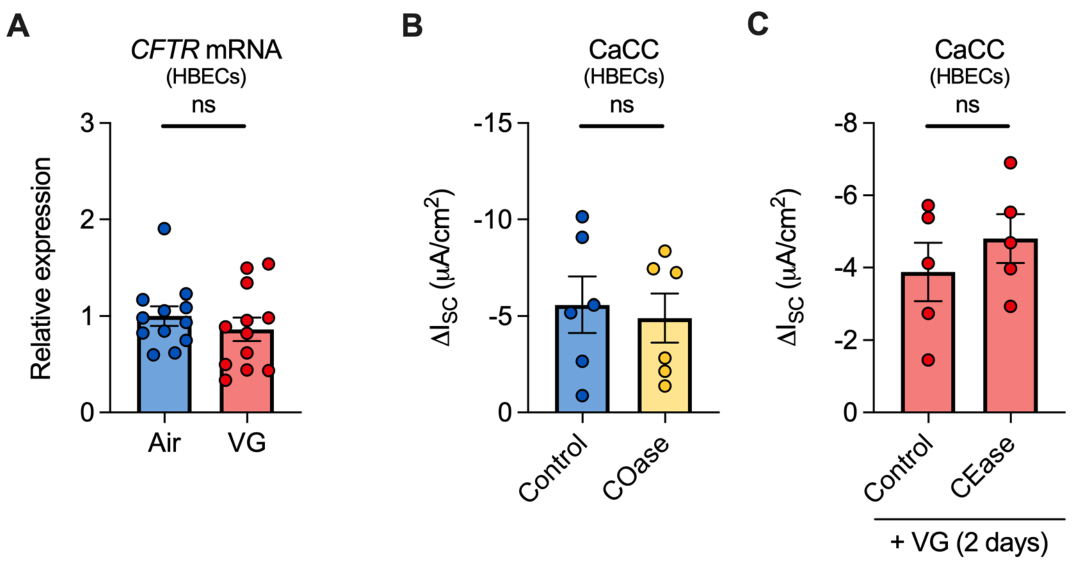

Supplementary Figure S1. (A) Exposure of HBECs to VG e-cig aerosols did not significantly affect *CFTR* mRNA expression levels after seven days. n = 12 lungs. **(B)** CaCC currents remain unchanged in HBECs acutely treated with cholesterol oxidase (COase, 1 U/mL, 30 min) compared to buffer only controls. n = 6 from 4 lungs. **(C)** Increasing membrane fluidity with cholesterol esterase (CEase, 0.2 U/mL, 30 min) did not significantly change CaCC currents in HBECs exposed to VG e-cig aerosols for two days. n =5 lungs. **Statistics**: Data are presented as mean ± SEM. ns = not significant. Data were analyzed by two-tailed t-test after assessing normality by Shapiro-Wilk.

Supplementary Table S1

Characteristics of never-smoker donor lungs used in this study.

**Donor lung characteristics**	**Age** (years)
**Combined** (n = 28)	
Median	25
Range	15 – 79
**Female** (n = 12)	
Median	25
Range	15 – 49
**Male** (n = 16)	
Median	25
Range	15 – 79
